# Cariprazine maintenance treatment during pregnancy – a case report

**DOI:** 10.3389/fpsyt.2024.1421395

**Published:** 2024-06-12

**Authors:** Róbert Herold, Tamás Tényi, Márton Herold, Tünde Tóth

**Affiliations:** ^1^ Department of Psychiatry and Psychotherapy, Medical School, University of Pécs, Pécs, Hungary; ^2^ Psychiatry Outpatient Department, Markhot Ferenc Teaching Hospital and Clinic, Eger, Hungary

**Keywords:** pregnancy, antipsychotics, cariprazine, schizophrenia, case report

## Abstract

Data on reproductive safety of recently approved newer antipsychotics are limited. Here, we report a case vignette of a patient with schizophrenia treated with cariprazine during pregnancy. The patient became pregnant unexpectedly while taking medication. As a result of shared decision-making, the patient and her psychiatrist decided to continue the treatment, which proved to be protective against relapse and had no adverse effect either on the course of pregnancy or on the health of the newborn. Cariprazine maintenance treatment during pregnancy was found to be safe in our case.

## Introduction

The majority of women treated for schizophrenia spectrum disorder are in their reproductive years, and are very likely to be prescribed antipsychotics, but data on the reproductive safety of these medications are quite scarce (1). This can be a significant problem especially if the patient is planning to have children or becomes pregnant while taking antipsychotics. In such situations, the patient and clinician must consider whether discontinuation or continuation of the antipsychotic poses the greater risk. The vast majority of guidelines agree that the beneficial effect of continuing the treatment outweighs the potential fetal risk. There is convincing evidence that cessation of antipsychotics leads to increased risk of relapse, maternal hospitalization, and subsequent separation of the mother and the newborn, which significantly affects the mother-infant bond (2). In addition to worsening maternal disturbance, untreated psychotic disorder is also associated with increased risk of neonatal health problems in the newborn (low birthweight, preterm delivery, small for gestational age, fetal distress, etc) (3).

However, for ethical reasons, there are no clinical trials to assess the safety of antipsychotics in pregnancy. Thus, the reproductive safety of these medications can only be established from case reports and large database studies (4). Antipsychotics may have teratogenic or toxic effects on the fetus. In addition to large database studies, there are a number of systematic reviews and meta-analyses as well, and these data generally suggest that antipsychotics do not increase the risk of congenital malformations. There appears to be no significant difference between first- and second-generation antipsychotics (SGA) (4). Recent results from a cohort study of more than 27 000 mothers exposed to antipsychotics from 6 countries suggest that antipsychotics are not significant teratogens overall (5). Newborns may develop withdrawal syndrome or neurological symptoms; however these symptoms usually resolve within a short time, and follow-up studies have found no differences in behavioural functioning or IQ (4). Most recently, no significant effect of antipsychotics was reported on neurobehavioral outcome among preschool-aged children who were exposed to SGAs prenatally (6). In conclusion, based on these data, antipsychotics do not generally appear to increase the risk of congenital malformations and poor pregnancy outcomes.

Nonetheless, data on reproductive safety of recently approved newer antipsychotics are even more limited, and case reports therefore play an important role in guiding prescribing decisions. One of the most recently approved antipsychotic drugs is cariprazine. Cariprazine is a dopamine partial agonist with the highest affinity to D3 receptors, but it is also partial agonist on D2 and 5-HT1a receptors (7). It has two main active metabolites (desmethyl and didesmethyl cariprazine), and due to its long half-time, it may take several weeks to wash out (8). In addition to its antipsychotic effects, it has a significant impact on negative symptoms with a favourable side effect profile (9). Here, we report a case vignette of a patient treated with cariprazine during pregnancy.

## Case report

Mrs G is a 23 years old Caucasian female being treated for a DSM-5 diagnosis of schizophrenia. It should be noted that her father was also treated for schizophrenia. Her illness began at the age of 16 with depressive symptoms. The patient received psyhotherapy. She was later treated with venlafaxine for the recurrence of depressive symptoms. She was hospitalized with the first psychotic episode at the age of 17. She was treated with haloperidol 15mg, but it was switched to olanzapine due to extrapyramidal side effects. The patient was discharged on olanzapine 15 mg. At age 18, psychotic relapse developed due to non-compliance with medication. Olanzapine was reinstated and escitalopram was added due to depressive symptoms. Psychotic symptoms resolved, however, Mrs G experienced affective and negative symptoms, decreased functionality, and significantly increased weight. For this reason, olanzapine was switched to cariprazine at a dose of 3 mg by cross-titration, and the escitalopram therapy was continued. Since then, the patient was characterized by a stable condition and good adherence. No repeated hospitalisation was necessary under ambulatory management. No change in maintenance treatment was required during this period. The patient’s body weight also decreased, her functionality improved, and she became more socially active. She had a relationship, moved away from her parents, and established an independent life.

In 2021, Mrs G became pregnant, which was discovered in the 6th week of pregnancy, and she reported to her psychiatrist 2 weeks later. Mrs G was counselled on pregnancy-related issues, emphasizing the risks of continuing or discontinuing antipsychotic treatment. A joint decision was made to continue the effective cariprazine maintenance treatment during pregnancy, but to discontinue escitalopram. The patient attended regular prenatal care, and her psychiatrist closely monitored for symptom recurrence and side effects. The dose of cariprazine was 3 mg throughout pregnancy. The patient remained stable, and did not experience any significant side effects during the pregnancy.

After an uneventful pregnancy, a spontaneous vaginal delivery occurred in April 2022 at 40th week. She gave birth to a mature, healthy baby girl weighing 2700 grams, with an Apgar score of 9/9. After informing the mother, a joint decision was made, and breast-feeding was not introduced due to antipsychotic medication. No pharmacological treatment was used for ablactation. The early neonatal period was normal, and mother and daughter were discharged home without complaints. Cariprazine treatment was continued after delivery, and venlafaxine 50 mg was added to help her through the challenging postpartum period. The patient remained in remission despite several stressful events (her father suffered from a psychotic decompensation; parents divorced). During these periods psychological support was added, and no change in medication was required. In addition to providing adequate care for the child, she also supported her father during the recovery period. According to the careful pediatric examinations during the more than two-years of follow-up after delivery, the child showed normal development and functioning without postnatal complications, and an appropriate attachment relationship developed between the child and the mother. The follow-up of the child continues with the close cooperation of the pediatrician and the psychiatrist.

## Discussion

To our knowledge, this is the first report of cariprazine use during pregnancy. Cariprazine treatment has been shown to be safe in our case. It was protective against relapse and had no adverse effect on the course of pregnancy. There were no discontinuation symptoms at all or other undesirable effects on the health of the newborn.

Cariprazine is a recently approved antipsychotic, and therefore limited data are available on its reproductive safety. To our knowledge, there is only one animal study on the subject, which suggests that cariprazine may inhibit sterol synthesis, and thus have an adverse effect on neurodevelopment (10). Similar results were reported on amiodarone, aripiprazole, fluoxetine, haloperidol, and trazodone (11). Although the results of animal studies are not translatable directly to human conditions, we cannot rule out that human subjects with certain specific genotypes may be susceptible to prolonged sterol-inhibiting effects (11). In our case early neurodevelopment was still intact at 24 months of follow-up, suggesting that sterol synthesis was not affected in Mrs G’s newborn. However, it should be noted that the patient reported her pregnancy in the eighth week of gestation, after the most vulnerable period for teratogenesis. Therefore, discontinuation of cariprazine treatment would not have reduced the risk of teratogenesis. Besides, most guidelines do not recommend antipsychotic switch, because it may increase the risk of relapse (4, 12). Instead, the continuation of the lowest effective dose of the same compound is the favourable strategy (4, 12). Although antipsychotics may cause withdrawal symptoms or neurological effects, it is also not advisable to reduce dose in the third trimester because of the higher risk of the recurrence of the symptoms of schizophrenia in the highly vulnerable perinatal period (4, 12). According to these recommendations cariprazine treatment continued without tapering the dose before delivery. The low-dose (3 mg) cariprazine kept the patient in remission during pregnancy and the postpartum period, and did not cause withdrawal or neurological symptoms in the newborn.

We should also emphasize the limitations of our case report. A significant weakness is that the neurodevelopment of the child was based on pediatric examintations. The use of neuropsychological tests would have provided a more precise description of the development. It is also an important limitation that our report represents only one case with a low dose cariprazine, which weakens the generalizability of our experiences.

In the absence of well controlled studies to assess the potentially adverse effects of antipsychotics during pregnancy, accumulating clinical experience and case reports have an important role to play in gaining better insight into the reproductive safety of antipsychotics. In the case of Ms. G cariprazine treatment had a favourable outcome, for both mother and infant. As our patients reflected on her treatment: *“The regular psychiatric and obstatric care and the minimal medication with no side effects ensured that I could maintain my mental balance during pregnancy and that my baby could be born healthy”*. We hope that our report can give a modest contribution to a better understanding of the safety of cariprazine in pregnancy.

## Data availability statement

The original contributions presented in the study are included in the article/supplementary material. Further inquiries can be directed to the corresponding author.

## Ethics statement

Written informed consent was obtained from the individual(s) for the publication of any potentially identifiable images or data included in this article.

## Author contributions

RH: Writing – review & editing, Writing – original draft, Conceptualization. TTé: Writing – review & editing, Conceptualization. MH: Writing – review & editing. TTó: Writing – original draft.
